# Thrombocytopenia in *Klebsiella pneumoniae* liver abscess: a retrospective study on its correlation with disease severity and potential causes

**DOI:** 10.3389/fcimb.2024.1351607

**Published:** 2024-03-18

**Authors:** Lulu Chen, Hongguang Wang, Hairui Wang, Yawen Guo, Zhihui Chang

**Affiliations:** Department of Radiology, Shengjing Hospital of China Medical University, Shenyang, China

**Keywords:** thrombocytopenia, Klebsiella pneumoniae liver abscess, disease severity, thrombophlebitis, bone marrow analysis

## Abstract

**Objective:**

Thrombocytopenia is commonly associated with infectious diseases and serves as an indicator of disease severity. However, reports on its manifestation in conjunction with *Klebsiella pneumoniae* liver abscess (KPLA) are scarce. The present study sought to elucidate the correlation between thrombocytopenia and KPLA severity and delve into the etiological factors contributing to the incidence of thrombocytopenia.

**Materials and methods:**

A retrospective analysis of the clinical data from patients with KPLA admitted between June 2012 and June 2023 was performed. Baseline characteristics, biochemical assessments, therapeutic interventions, complications, and clinical outcomes were compared between patients with and without thrombocytopenia. To investigate the potential etiologies underlying thrombocytopenia, the association between platelet count reduction and thrombophlebitis was examined, with a particular focus on platelet consumption. Furthermore, bone marrow aspiration results were evaluated to assess platelet production anomalies.

**Results:**

A total of 361 KPLA patients were included in the study, among whom 60 (17%) had concurrent thrombocytopenia. Those in the thrombocytopenia group exhibited significantly higher rates of thrombophlebitis (p = 0.042), extrahepatic metastatic infection (p = 0.01), septic shock (p = 0.024), admissions to the intensive care unit (p = 0.002), and in-hospital mortality (p = 0.045). Multivariate analysis revealed that thrombocytopenia (odds ratio, 2.125; 95% confidence interval, 1.114–4.056; p = 0.022) was independently associated with thrombophlebitis. Among the thrombocytopenic patients, eight underwent bone marrow aspiration, and six (75%) had impaired medullar platelet production. After treatment, 88.6% of thrombocytopenic patients (n = 47) demonstrated recovery in their platelet counts with a median recovery time of five days (interquartile range, 3–6 days).

**Conclusions:**

Thrombocytopenia in patients with KPLA is indicative of increased disease severity. The underlying etiologies for thrombocytopenia may include impaired platelet production within the bone marrow and augmented peripheral platelet consumption as evidenced by the presence of thrombophlebitis.

## 1 Introduction

In recent decades, *Klebsiella pneumoniae* (*K. pneumoniae*) has emerged as the primary pathogen for community-acquired liver abscesses in many Asian countries, surpassing Gram-positive cocci (Luo et al., 2016). Its hypermucoviscous phenotype is associated with a heightened risk of extrahepatic metastatic infections in *K. pneumoniae* liver abscess (KPLA), notably affecting the lungs, eyes, or brain (Fang et al., 2007; Tan et al., 2020). A significant occurrence of regional thrombophlebitis in hepatic veins has been observed in KPLA cases in contrast with other liver abscess etiologies (Alsaif et al., 2011).

Thrombocytopenia is characterized by platelet counts below 100×10^9/L and is prevalent among septic patients (Shannon, 2021). This condition has been linked to disease severity, including increased bleeding tendencies, higher transfusion requirements, and in-hospital death (Zhou et al., 2020). To date, there have been no studies investigating the relationship between thrombocytopenia and disease severity in KPLA. Beyond their primary role in hemostasis, platelets are integral to the immune system (Morrell et al., 2014). Their involvement in the immune response against infections, termed immunothrombosis, has garnered significant attention (Engelmann and Massberg, 2013). Platelets can adhere to bacteria, triggering a cascade of events that foster thrombus formation and ensuring its confinement to the injury site (Cox, 2023). In thrombophlebitis, platelets are activated and play an immunomodulatory role, leading to increased platelet consumption, which may be associated with thrombocytopenia in patients with KPLA. In addition, impaired bone marrow platelet production may be another potential cause of thrombocytopenia during infection.

Therefore, the objective of our study was to examine the relationship between thrombocytopenia and KPLA severity and to explore the underlying causes from the perspectives of platelet consumption and bone marrow platelet production.

## 2 Methods

### 2.1 Study design and approval

This retrospective study received approval from the Ethics Committee of the Shengjing Hospital of China Medical University (No.2022PS146K) and adhered to the principles outlined in the Declaration of Helsinki. Given the study’s retrospective nature, the requirement for informed consent was waived.

### 2.2 Microbiota identification

Bacterial identification and antimicrobial susceptibility testing were conducted using the VITEK-compact automatic microbiological analysis system (France BioMérieux) and interpreted according to guidelines established by the Clinical and Laboratory Standards Institute (CLSI). Phenotypic confirmation of ESBL was performed using the double-disk diffusion method in our clinical microbiology laboratories, following guidelines established by the Clinical and Laboratory Standards Institute(Kim et al., 2019).

### 2.3 Patient selection

Patients diagnosed with KPLA between January 2012 and June 2023 were retrospectively reviewed. Inclusion criteria were as follows: (1) diagnosis of pyogenic liver abscess (PLA) based on contrast-enhanced computed tomography (CECT) scan upon admission; and (2) isolation of *K. pneumoniae* strains from either pus or blood culture. Patients were excluded if they: (1) were under 18 years of age; (2) had hematologic disease involving platelets, such as myelodysplastic syndrome; and (3) lacked essential laboratory results.

### 2.4 Data collection

Data were systematically extracted from the medical record database and included demographic details (age and sex), clinical information (symptoms, underlying conditions, laboratory findings, duration of hospital stay, treatment regimen, complications, intensive care unit (ICU) admissions, and in-hospital mortality), and results from bone marrow aspirations.

### 2.5 Variable definition

Thrombocytopenia was characterized by platelet counts falling below 100×10^9/L, which is a criterion informed by established literature sources (Menard et al., 2019). Extrahepatic metastatic infection (EMI) was identified as the occurrence of septic pulmonary embolism, endophthalmitis, brain abscesses, meningitis, muscle abscesses, and necrotizing fasciitis during the same admission confirmed by clinical records or imaging (Siu et al., 2012). Thrombophlebitis was recognized by hypodense filling defects in contrast-enhanced hepatic veins or their branches, including the inferior vena cava (Alsaif et al., 2011). Poor glycemic control was marked by HbA1c level of ≥6.5% or fasting blood glucose level of ≥7 mmol/L. Biliary tract diseases were considered to be benign conditions, such as biliary stones, cholecystitis, or cholangitis, irrespective of any prior surgical interventions (Wang et al., 2021a). Digestive system tumors included hepatobiliary pancreatic or gastrointestinal malignancies regardless of surgical history (Wang et al., 2021b). Hemophagocytosis was diagnosed using histological findings where activated macrophages engulfed other cells in bone marrow samples (Buyse et al., 2010). Lastly, platelet recovery was marked by two consecutive platelet counts of ≥100×10^9/L during a patient’s hospital stay (Menard et al., 2019). The definition of impaired medullar platelet production is the absence of platelet-producing megakaryocyte observed in bone marrow smear, excluding inadequate bone marrow sampling.

### 2.6 Statistical analysis

Data entry and processing were conducted using R, a language and environment for statistical computing (R Foundation for Statistical Computing, Vienna, Austria) and SPSS version 26 software (IBM Co, Armonk, NY, USA). The normality of numerical variable distribution was tested using the Shapiro–Wilk test. Continuous variables with a normal distribution were expressed as the mean ± standard deviation and compared using unpaired t tests. Continuous variables with a skewed distribution were expressed as the median (quartile distance) and compared using the Mann–Whitney U test. Categorical variables were presented as frequencies and percentages and analyzed using the χ2 test or Fisher’s exact test. Thrombocytopenia was evaluated using binary variables. Logistic regression was used for multivariate analyses to predict thrombophlebitis outcomes. Baseline variables that were considered clinically relevant or showed a univariate relationship with the outcome were entered into the multivariate logistic regression analysis model. Variables for inclusion were carefully chosen given the number of events available to ensure the parsimony of the final model. All p-values of <0.05 were considered statistically significant.

## 3 Results

### 3.1 Baseline characteristics of KPLA patients

**Figure 1:**
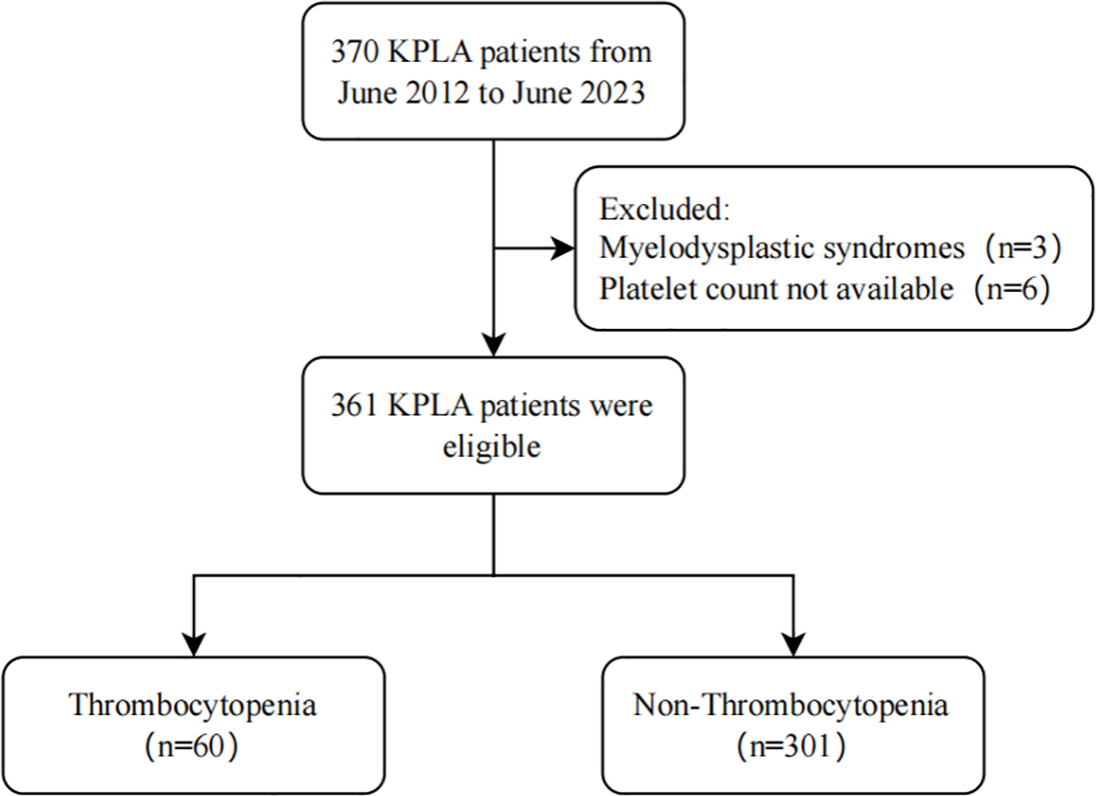
Flow diagram depicting patient selection.

During the study period, 370 KPLA patients (**Figure 1**) were assessed and 361 subjects were included in the final analysis. Patients were grouped based on their platelet counts at admission. Their characteristics are presented in **Table 1**.

**Table 1:** General characteristics of the KPLA patients at admission.

Variables	Total (n = 361)	Thrombocytopenia (n = 60)	Non-Thrombocytopenia (n = 301)	p
Demographic data				
Age, median (IQR)	59 (50, 68)	59 (49.75, 65)	59 (50, 68)	0.792
Male (sex)	208 (57.6)	35 (58.3)	173 (57.5)	1
Comorbidities				
Diabetes mellitus	254 (70.4)	45 (75)	209 (69.4)	0.48
Poor glycemic control	216 (59.8)	39 (65)	177 (58.8)	0.453
Hypertension	65 (18)	10 (16.7)	55 (18.3)	0.911
Hepatic dysfunction	179 (49.6)	39 (65)	140 (46.5)	**0.013**
Cirrhosis	12 (3.3)	3 (5)	9 (3)	0.429
Biliary tract disease	103 (28.5)	18 (30)	85 (28.2)	0.905
Digestive system tumors	25 (6.9)	2 (3.3)	23 (7.6)	0.4
Gastrointestinal surgery history	35 (9.7)	8 (13.3)	27 (9)	0.421
Symptoms				
Fever	326 (90.3)	57 (95)	269 (89.4)	0.268
Chills	163 (45.2)	25 (41.7)	138 (45.8)	0.651
Abdominal pain	96 (26.6)	14 (23.3)	82 (27.2)	0.641
Vomiting and diarrhea	37 (10.2)	10 (16.7)	27 (9)	0.118
Jaundice	10 (2.8)	4 (6.7)	6 (2)	0.066
Imaging features				
Location of liver abscess				0.453
Right lobe	273 (75.6)	49 (81.7)	224 (74.4)	
Left lobe	63 (17.5)	9 (15)	54 (17.9)	
Both lobes	25 (6.9)	2 (3.3)	23 (7.6)	
Multiple lesions	93 (25.8)	14 (23.3)	79 (26.2)	0.757
Length (mm)	65 (51, 85)	58 (44.75, 75.5)	68 (53, 85)	**0.029**
Containing gas	70 (19.4)	20 (33.3)	50 (16.6)	**0.005**
Laboratory findings, median (IQR)				
WBC (10^9/L)	10.84 (8.33, 14.04)	10.1 (7.79, 14.04)	11 (8.4, 14.04)	0.207
Neu (10^9/L)	8.9 (6.3, 11.85)	8.69 (6.57, 12.46)	8.9 (6.2, 11.8)	0.761
Ly (10^9/L)	1.1 (0.75, 1.5)	0.8 (0.5, 1.13)	1.2 (0.8, 1.6)	**< 0.001**
PLT (10^9/L)	202 (127, 322)	54.5 (30.5, 76)	238 (168, 349)	**< 0.001**
MPV (fL)	9.59 (8.2, 10.6)	10.75 (9.97, 12.22)	9.3 (8, 10.3)	**< 0.001**
Hb (g/L)	116 (103, 129)	118 (105.25, 132)	116 (103, 127)	0.326
PT (s)	13.6 (12.7, 14.8)	13.5 (12.2, 14.5)	13.6 (12.8, 15)	0.059
INR	1.2 (1.1, 1.38)	1.2 (1, 1.3)	1.2 (1.1, 1.4)	**0.049**
D-Dimor (μg/L)	1021 (554, 2201)	2333.5 (810.25, 5019)	951 (481, 1763)	**< 0.001**
Albumin (U/L)	29.9 (26.3, 33.6)	27.15 (24.1, 30.33)	30.6 (26.7, 34.1)	**< 0.001**
ALT (U/L)	52 (30, 92)	82 (45, 138.75)	48 (28, 85)	**< 0.001**
AST (U/L)	36 (22, 60)	57.37 (33.25, 104.25)	32 (20, 56)	**< 0.001**
TBil (μmol/L)	13.6 (9, 20.3)	19.85 (13.23, 36.5)	12.2 (8.8, 18.8)	**< 0.001**
Creatinine (μmol/L)	62 (50.1, 77.4)	84.25 (66.07, 153)	59.1 (48.8, 72)	**< 0.001**
CRP (mg/L)	149 (110, 200)	186.8 (132.96, 286.58)	144.99 (101, 185)	**< 0.001**
ESBL, n (%)	23 (6.4)	2 (3.3)	21 (7)	0.394

Bold values are statistically significant (p < 0.05); Data are shown as median (IQR) or n (%); IQR, interquartile range. Fever means body temperature of >38.5°C; Length, maximum diameter of an abscess; WBC, white blood cell; Neu, neutrophils; Ly, lymphocytes; PLT, platelet counts; MPV, mean platelet volume; Hb, hemoglobin; PT, prothrombin time; INR, International normalized ratio; ALT, alanine aminotransferase; AST, aspartate aminotransferase; TBIL, total bilirubin; CRP, C-reactive protein; ESBL, extended-spectrum beta-lactamase.

### 3.2 Thrombocytopenia indicates disease severity in KPLA

The thrombocytopenia group exhibited more severe inflammatory responses, liver and kidney function impairment, and coagulation activation (**Table 1**).

KPLA management primarily involved a combination of antibiotics and percutaneous drainage (92%), with no significant differences observed in the treatment methods between the two groups (**Table 2**, p = 0.413).

**Table 2:** Treatment, complications, and outcome variables in KPLA patients.

Variables	Total (n = 361)	Thrombocytopenia (n = 60)	Non-Thrombocytopenia (n = 301)	p
**Treatment**				0.413
Antibiotics alone	29 (8)	7 (11.7)	22 (7.3)	
Antibiotics + Percutaneous drainage	331 (91.7)	53 (88.3)	278 (92.4)	
Antibiotics + Surgical resection	1 (0.3)	0 (0)	1 (0.3)	
Complication				
Abscess rupture	13 (3.6)	1 (1.7)	12 (4)	0.703
Respiratory failure	11 (3)	4 (6.7)	7 (2.3)	0.092
Thrombophlebitis	97 (26.9)	23 (38.3)	74 (24.6)	**0.042**
EMI	73 (20.2)	20 (33.3)	53 (17.6)	**0.01**
Septic shock	15 (4.2)	6 (10)	9 (3)	**0.024**
**Length of hospital stay**, median (IQR)	11 (8, 17)	13.5 (8, 21.25)	11 (8, 17)	0.122
**ICU occupation**	20 (5.5)	9 (15)	11 (3.7)	**0.002**
**In-hospital mortality**	9 (2.5)	4 (6.7)	5 (1.7)	**0.045**

Data are represented as No. (%) unless otherwise indicated; IQR, interquartile range. Bold values are statistically significant (p < 0.05), ICU, intensive care unit.

Thrombocytopenic patients presented with a higher incidence of complications, including thrombophlebitis (38.3% vs. 24.6%; p = 0.042), EMI (33.3% vs. 17.6%; p = 0.042), and septic shock (10% vs. 3%; p = 0.024) when compared to patients in the non-thrombocytopenic group. Notably, patients with thrombocytopenia were more likely to experience ICU admission (15% vs. 3.7%; p = 0.002) and in-hospital mortality (6.7% vs. 1.7%; p = 0.045) during their hospitalization in comparison to patients without thrombocytopenia.

### 3.3 Thrombocytopenia as an independent correlated factor for thrombophlebitis

Platelets may be significantly activated and consumed during the development of immunothrombosis (Engelmann and Massberg, 2013), and hepatic venous thrombosis in KPLA is a distinctive manifestation of immune thrombophlebitis. Therefore, univariate analyses and multivariate logistic regression analyses were performed to investigate the association between thrombocytopenia and thrombophlebitis (**Tables 3**, **4**). Thrombocytopenia (odds ratio [OR], 2.125; 95% confidence interval (CI), 1.114–4.056; p = 0.022) was an independent correlated factor associated with thrombophlebitis in addition to poor glycemic control (OR, 1.985; 95% CI, 1.185–3.324; p = 0.009).

**Table 3:** Clinical characteristics of KPLA patients with or without thrombophlebitis.

Variables	Total (n = 361)	Thrombophlebitis (n = 97)	Non-Thrombophlebitis (n = 264)	p
Demographic data				
Age, median (IQR)	59 (50, 68)	57 (48, 64)	60 (51, 69)	**0.044**
Male (sex)	153 (42.4)	31 (32)	122 (46.2)	**0.021**
Comorbidities				
Diabetes mellitus	254 (70.4)	72 (74.2)	182 (68.9)	0.398
Poor glycemic control	216 (59.8)	70 (72.2)	146 (55.3)	0.006
Hypertension	65 (18)	15 (15.5)	50 (18.9)	0.544
Hepatic dysfunction	179 (49.6)	54 (55.7)	125 (47.3)	0.199
Cirrhosis	12 (3.3)	4 (4.1)	8 (3)	0.741
Biliary tract disease	103 (28.5)	25 (25.8)	78 (29.5)	0.567
Digestive system tumors	25 (6.9)	11 (11.3)	14 (5.3)	0.077
Gastrointestinal surgery history	35 (9.7)	12 (12.4)	23 (8.7)	0.4
Laboratory findings, median (IQR)				
Thrombocytopenia	60 (16.6)	23 (23.7)	37 (14)	**0.042**
WBC (10^9/L)	10.84 (8.33, 14.04)	10.84 (7.8, 15.4)	10.86 (8.58, 13.7)	0.932
Neu (10^9/L)	8.9 (6.3, 11.85)	9 (5.7, 13.5)	8.8 (6.45, 11.5)	0.611
Ly (10^9/L)	1.1 (0.75, 1.5)	1.1 (0.7, 1.44)	1.19 (0.8, 1.59)	0.123
MPV (fL)	9.59 (8.2, 10.6)	9.6 (8.6, 10.8)	9.4 (8.2, 10.53)	0.079
Hb (g/L)	116 (103, 129)	119 (109, 129)	115 (101, 128)	0.098
PT (s)	13.6 (12.7, 14.8)	13.3 (12.7, 14.8)	13.7 (12.8, 14.81)	0.234
INR	1.2 (1.1, 1.38)	1.2 (1.1, 1.36)	1.2 (1.1, 1.38)	0.262
D-Dimor (μg/L)	1021 (554, 2201)	1070 (501, 2775)	981 (562, 2064)	0.482
Albumin (U/L)	29.9 (26.3, 33.6)	30.2 (26.3, 33.6)	29.9 (26.37, 33.52)	0.721
ALT (U/L)	52 (30, 92)	57 (32, 98)	50 (28.75, 89.25)	0.13
AST (U/L)	36 (22, 60)	43 (23, 70)	34 (20.75, 58.25)	0.165
TBil (μmol/L)	13.6 (9, 20.3)	14.9 (10.1, 21.1)	12.9 (9, 19.77)	0.3
Creatinine (μmol/L)	62 (50.1, 77.4)	67.9 (52.3, 83.1)	60.95 (49.08, 74.2)	**0.015**
CRP (mg/L)	149 (110, 200)	155.75 (106, 209)	145.88 (111.68, 195.6)	0.552
ESBL	23 (6.4)	5 (5.2)	18 (6.8)	0.741
**Length of hospital stay**, median (IQR)	11 (8, 17)	13 (9, 22)	10 (8, 17)	**0.004**
Complication				
Abscess rupture	13 (3.6)	3 (3.1)	10 (3.8)	1
Respiratory failure	11 (3)	7 (7.2)	4 (1.5)	**0.01**
EMI	73 (20.2)	47 (48.5)	26 (9.8)	**< 0.001**
Septic shock	15 (4.2)	6 (6.2)	9 (3.4)	0.244
**ICU occupation**	20 (5.5)	5 (5.2)	15 (5.7)	1
**In-hospital mortality**	9 (2.5)	1 (1)	8 (3)	0.454

Data are presented as No. (%) unless otherwise indicated; IQR, interquartile range. Bold values mean statistical significance (p < 0.05). Fever means body temperature of >38.5°C; Length, maximum diameter of an abscess; WBC, white blood cell; Neu, neutrophils; Ly, lymphocytes; PLT, platelet counts; MPV, mean platelet volume; Hb, hemoglobin; PT, prothrombin time; INR, International normalized ratio; ALT, alanine aminotransferase; AST, aspartate aminotransferase; TBIL, total bilirubin; Cr, creatinine; CRP, C-reactive protein; ICU, intensive care unit. ESBL, extended-spectrum beta-lactamase. "^" indicates exponentiation, "10^9/L", ten raised to the power of nine per liter.

**Table 4:** Multivariable logistic regression analysis for thrombophlebitis.

Variables	Multivariate Analysis		
	OR	95%CI	P value
Age	0.992	(0.974-1.011)	0.406
Male (sex)	1.659	(0.979-2.810)	0.06
**Poor glycemic control**	1.985	(1.185-3.324)	**0.009**
Creatinine	0.998	(0.994-1.002)	0.359
**Thrombocytopenia**	2.125	(1.114-4.056)	**0.022**

Bold values are statistically significant (p < 0.05).

### 3.4 Bone marrow aspiration reveals that KPLA patients with thrombocytopenia exhibit impaired medullary platelet production

Bone marrow samples were collected and analyzed from a subset of 19 patients to further explore the possible causes of thrombocytopenia. Thrombocytopenia was evident in eight cases (42%). Details of bone marrow findings are presented in **Table 5**. The majority of samples (n = 18, 94.7%) were obtained from the posterior superior iliac spine.

**Table 5:** Results of bone marrow aspirates.

Bone marrow findings	Thrombocytopenia (n = 8)	Non-Thrombocytopenia (n = 11)
Puncture site		
Posterior superior iliac spine	7 (87.5)	11 (100)
Sternum	1 (12.5)	0 (0)
Megakaryocytes counts		
Normal	4 (50)	7(63.6)
Increased	2 (25)	3 (27.3)
Decreased	2 (25)	1(9.1)
**Impaired medullar platelet production**	6 (75)	0 (0)
**Hemophagocytosis**	4 (50)	3 (27.3)
**Giant platelet**	0 (0)	1 (9.1)
**Infection symptom**	6 (75)	7 (63.6)
**Without cytological abnormality**	0 (0)	3 (27.3)

Data are shown as n (%).

**Figure 2:**
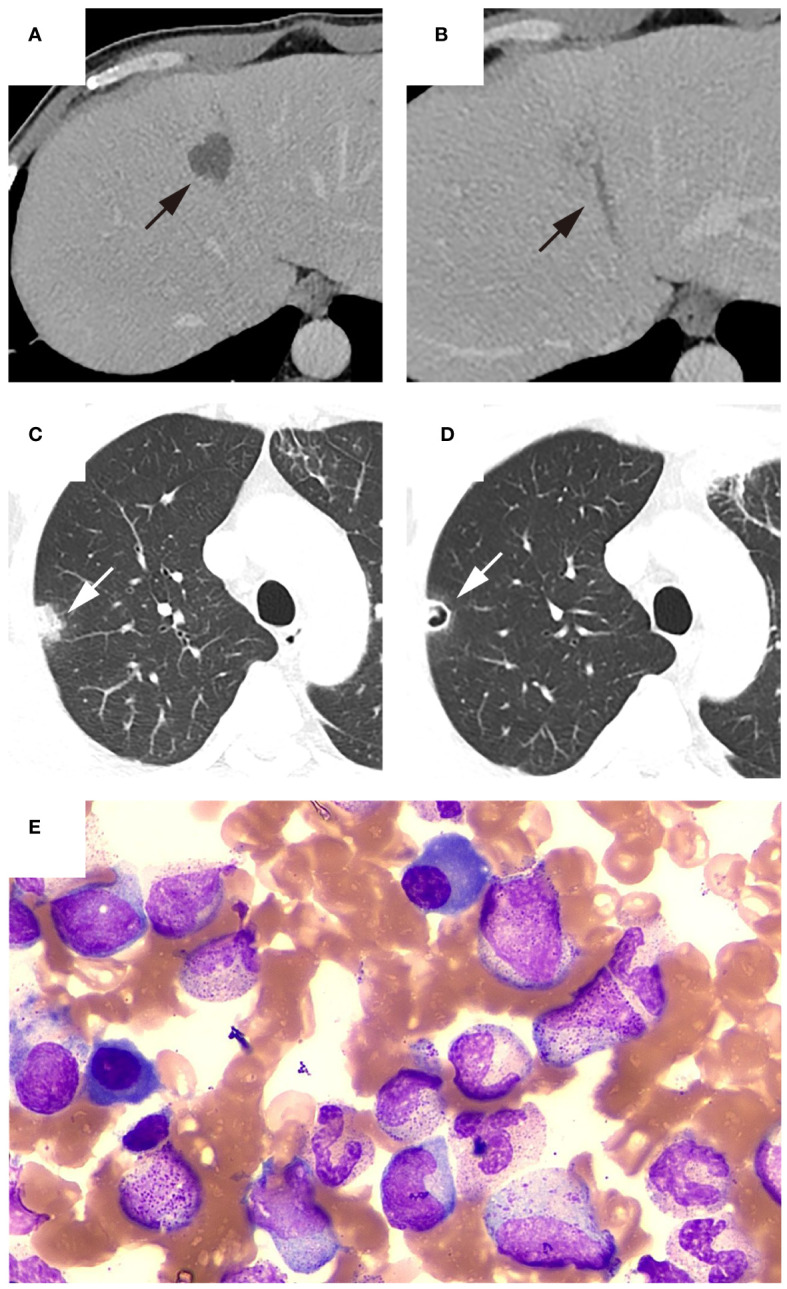
Representative patient with thrombocytopenia. The patient was admitted to the hospital with a high fever and chills lasting for two days. CECT showed (**(A)**, black arrow) liver abscess with (**(B)**, black arrow) thrombophlebitis of middle hepatic vein, and (**(C)**, white arrow) chest CT revealed a nodule in the right upper lobe of the lung. A follow-up chest CT five days after admission showed (**(D)**, white arrow) the presence of a new cavity within previously identified lesion in the right upper lobe. Laboratory tests indicated a platelet count of 36×10^9/L. Bone marrow aspiration was performed to determine the cause of thrombocytopenia. **(E)** Bone marrow smear showed megakaryocyte hyperplasia and no thromocytogenic megakaryocyte, indicating poor platelet production. Subsequently, blood culture results were positive for Klebsiella pneumoniae. Blood glucose monitoring after admission revealed poor glycemic control. During hospitalization, the patient received antibiotics and hypoglycemic treatment and the platelet count recovered to 103×10^9/L three days after admission. The patient was discharged after 21 days of hospitalization due to reduced abscess size and significant decrease in inflammatory markers.

**Figure 3:**
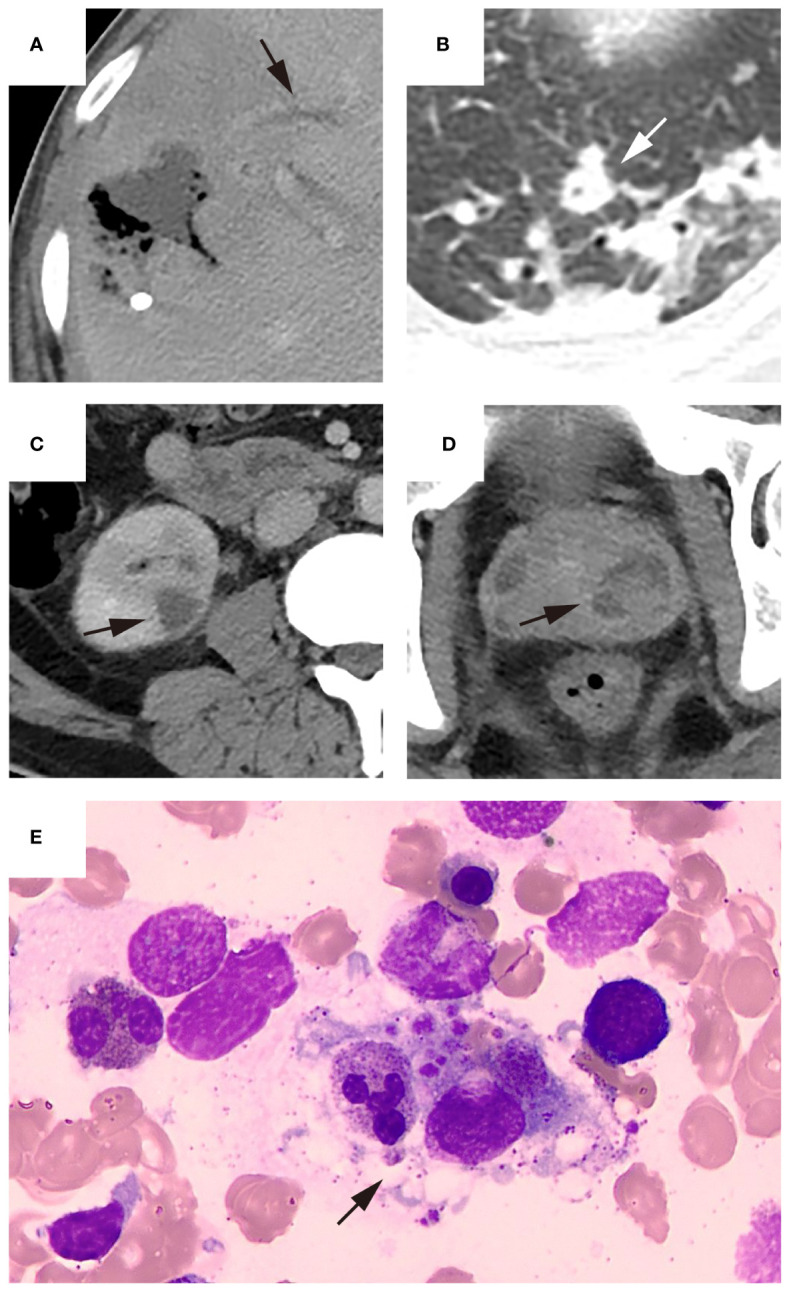
Representative patient who underwent bone marrow puncture after percutaneous drainage. The patient was admitted due to the discovery of a liver abscess at an external hospital. Upon admission, the platelet count was 104×10^9/L and hemoglobin level was 95 g/L. The patient underwent percutaneous drainage two days after admission, and bacterial culture of the drainage fluid was positive for Klebsiella pneumoniae. Imaging examination after puncture revealed (**(A)**, black arrow) thrombophlebitis of the middle hepatic vein, (**(B)**, white arrow) a small abscess in the left lung and (**(C)**, black arrow) multiple abscesses in the right kidney and (**(D)**, black arrow) prostate. Due to the patient’s history of previous malaria infection, low platelet count, and presence of anemia symptoms, bone marrow puncture examination performed three days after admission showed (**(E)**, black arrow) normal platelet production and the presence of hemophagocytosis. The patient’s platelet count increased to 156×10^9/L upon reexamination. After 12 days of hospitalization, the patient’s condition improved enough to be discharged.

**Figure 4:**
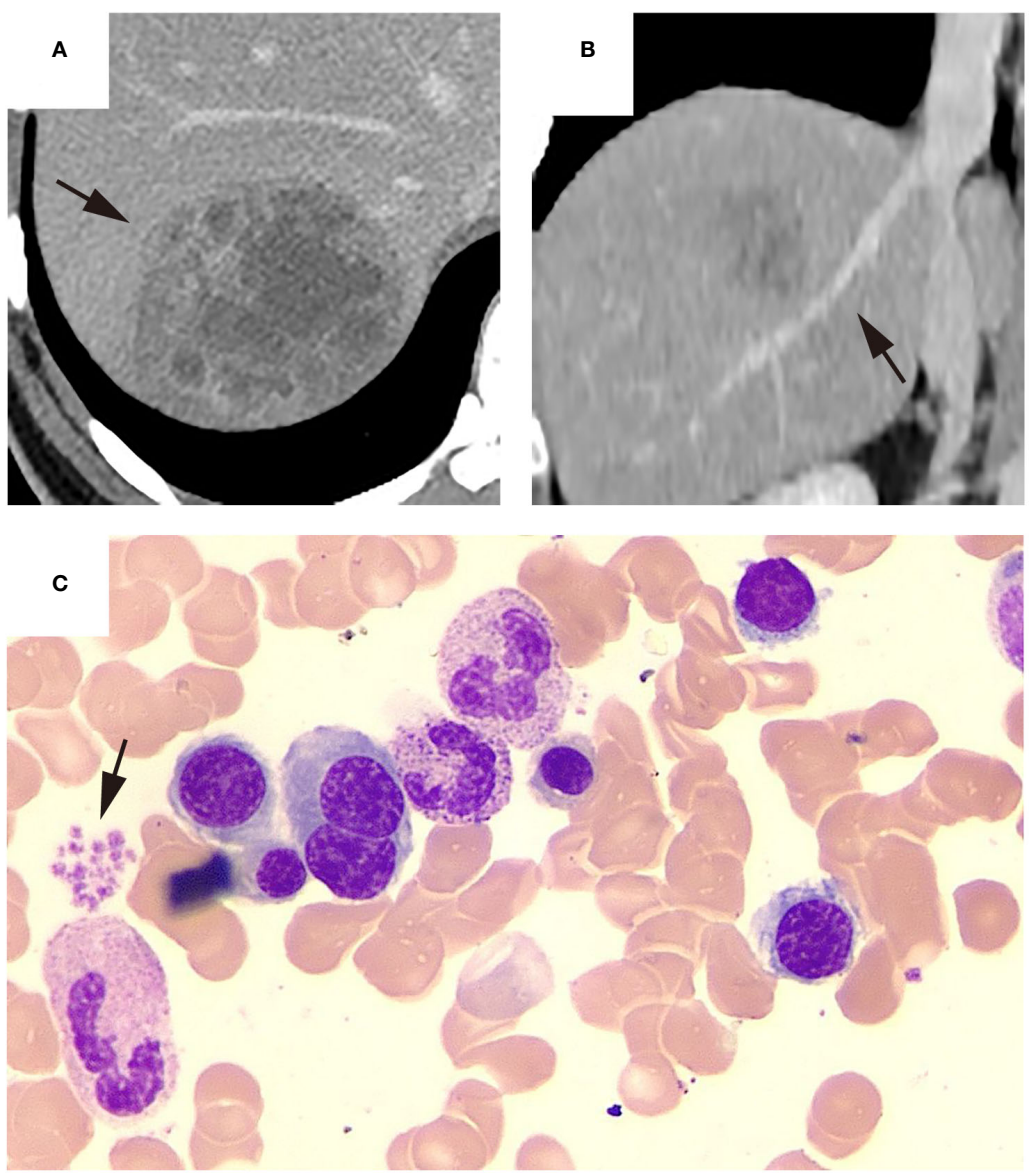
Representative KPLA patient without thrombocytopenia. The patient was admitted after experiencing a fever for 9 days, with a platelet count of 316 ×10^9/L at the time of admission. Upon admission, the patient underwent an abdominal CECT examination, which revealed (**(A)**, black arrow) a liver abscess in the right lobe and (**(B)**, black arrow) no filling defects in the hepatic vein. Subsequently, the patient underwent a percutaneous drainage procedure, where the drainage fluid was cultured, revealing a positive result for Klebsiella pneumoniae. During hospitalization, the patient underwent a bone marrow aspiration examination. The bone marrow smear showed the relative abundance of megakaryocytes and other cell lineages appeared normal and (**(C)**, black arrow) platelets were observed to aggregate in piles. After 15 days of hospitalization, the patient’s condition improved enough to be discharged.

Among the eight patients with thrombocytopenia, the majority (n = 6, 75%) exhibited impaired medullar platelet production, of whom two patients also demonstrated decreased megakaryocyte counts. Four patients with thrombocytopenia developed hemophagocytosis. Bone marrow smear and CT findings from representative patients with KPLA are shown in **Figures 2**–**4**.

### 3.5 Treatment and platelet recovery in KPLA patients with thrombocytopenia

Platelet counts were analyzed before discharge in thrombocytopenic patients, with the exception of three patients who were not retested for platelet counts prior to discharge and four patients who died during hospitalization (**Table 6**).

**Table 6:** Platelet recovery status of KPLA patients with thrombocytopenia before discharge.

Variables	Thrombocytopenia (n = 53)
**Age**, median (IQR)	59 (48.5, 65)
Sex	
Female	21 (39.6)
Male	32 (60.4)
Treatment	
Antibiotic	53 (100)
Percutaneous drainage	47 (88.7)
Platelet transfusion	5 (9.4)
rhTPO	2 (3.8)
**PLT on admission**, median (IQR)	55 (30, 76.5)
**PLT before discharge**, median (IQR)	268 (176,340)
**Platelet recovery**	47 (88.7)
**No platelet recovery**	6 (11.3)
Clinical symptom relief	3 (5.7)
Discharge against medical advice	3 (5.7)
**Median days of platelet recovery**, median (IQR)	5 (3, 6)

Data are presented as No. (%) unless otherwise indicated; IQR, interquartile range. PLT, platelet count; rhTPO, recombinant human thrombopoietin; Median days of platelet recovery, median time from thrombocytopenia onset at admission to platelet recovery.

Only 9.4% (n = 5) of the thrombocytopenia cases received platelet transfusions and 3.8% (n = 2) were administered recombinant human thrombopoietin (rhTPO). After treatment, 88.7% (n = 47) of thrombocytopenic patients demonstrated recovery in their platelet counts, with a median time of five days (IQR, 3–6 days) from admission to platelet recovery. Six patients did not recover before discharge. Among them, three patients were discharged against medical advice and the remaining three patients were discharged due to alleviation of clinical symptoms.

## 4 Discussion

Thrombocytopenia commonly coexists with various infectious diseases, such as sepsis (Thiery-Antier et al., 2016), community-acquired pneumonia (CAP) (Cunha and Hage, 2011), COVID-19(Zhao et al., 2020), and hepatitis B (Jiang et al., 2019), with an incidence ranging from 2% to 20.5%. It is often indicative of a more severe illness and a poorer clinical outcome, which is further supported by a recent study on PLA (Li et al., 2023). However, liver abscesses caused by *K. pneumoniae* commonly show distinct imaging features and clinical presentations compared to those caused by other bacteria, with higher rates of thrombophlebitis and extrahepatic metastatic infection (Chung et al., 2007; Alsaif et al., 2011). This is mainly attributed to its unique virulence factors, including up to four siderophore systems for iron acquisition, increased capsule production, K1 and K2 capsule types, and the colibactin toxin, along with antibiotic resistance (Fasciana et al., 2019; Choby et al., 2020). Therefore, our study is the first to specifically focus on the association between thrombocytopenia and KPLA severity and further analyzed the etiology of thrombocytopenia.

The present investigation corroborates the existing literature by revealing that KPLA patients with thrombocytopenia exhibit significantly enhanced inflammatory responses, heightened hepatic and renal dysfunction, and greater occurrence of adverse outcomes, including increased mortality. The etiology of this association may stem from the compromised immune system’s capabilities due to diminished platelet counts, which in turn exacerbates infection symptoms. In contrast, the progression of the infection itself may promote the degradation of platelets and hinder their synthesis (Klinger and Jelkmann, 2002; Zhou et al., 2020). Despite the intricacies of the relationship between thrombocytopenia and disease gravity, lower platelet counts portend a shift towards a more critical state, thereby demanding increased clinical vigilance. Interestingly, thrombocytosis has also been found to be a prognostic marker for negative outcomes in infectious diseases as detailed by Prina et al., who note its association with poor prognoses in CAP patients, including severe complications like pleural effusion and empyema (Prina et al., 2013). Further findings suggest a significant link between both thrombocytosis and thrombocytopenia and a higher risk of mortality in septic patients with leukocytosis (Bakey et al., 2013). This evidence highlights the influence of platelet count variations on the course and seriousness of sepsis, emphasizing the imperative for comprehensive research on the pathophysiological mechanisms involved.

The presence of a filling defect in the hepatic vein adjacent to a liver abscess is a distinctive and frequent radiographic manifestation of KPLA that is characterized as thrombophlebitis (Perez-Cruet et al., 1993; Hanazaki et al., 2001). Thrombophlebitis is theorized to arise from platelet activation subsequent to KP infection, triggering the coagulation cascade, which is a process known as immunothrombosis. It is feasible for immunothrombosis to occur at sites of infection, a phenomenon extensively documented in the pulmonary regions of COVID-19 patients (Kvietys et al., 2021). The hepatic vein near the abscess is particularly vulnerable to this condition, epitomizing a classic case of immunothrombosis. The present study’s multivariate analysis substantiated that thrombocytopenia is independently correlated with thrombophlebitis. This supports the conjecture that there is a significant activation and subsequent consumption of platelets during the process of immunothrombosis associated with KPLA (Engelmann and Massberg, 2013; Cox, 2023).

Our prior research has established poor glycemic control as a pivotal factor in KPLA propagation to extrahepatic metastatic infection (Wang et al., 2022). The present study further revealed that poor glycemic control is intricately linked to thrombophlebitis in an independent manner. It has been documented by Lee et al. that diabetic individuals with HbA1c levels of ≥9% are predisposed to enhanced platelet activation, consequently exhibiting elevated occurrences of hepatic venous thrombophlebitis (Lee et al., 2018). The precise mechanisms by which diabetes escalates the risk of thrombophlebitis remain to be fully understood. However, this phenomenon can be attributable to heightened platelet reactivity or irregular activation within the coagulation pathway. Moreover, the escalation in platelet activation and subsequent immunothrombosis may induce increased peripheral platelet consumption, which can then accelerate the onset of thrombocytopenia.

In the present investigation, comprehensive data on patients with KPLA were collected from various departments. Notably, a subset of these patients was subjected to bone marrow aspiration tests within the infectious diseases department, thus enabling the examination of the potential link between thrombocytopenia and suppressed bone marrow platelet synthesis. The study findings point towards compromised bone marrow platelet production as the primary contributor to thrombocytopenia, which is presumably connected to the septic condition incited by KPLA. Sepsis-induced inflammatory cytokines can compromise bone marrow stem cells, reducing megakaryocyte levels and impairing platelet production (Frydman et al., 2023). Additionally, hemophagocytosis, which is a condition marked by macrophages consuming blood cells, was observed in half of the thrombocytopenic KPLA patients (Zoller et al., 2011). This phenomenon can occur in severe inflammatory conditions, including sepsis (Ito et al., 2006; Castillo and Carcillo, 2009), influenza (Ando et al., 2006), and malaria (Zoller et al., 2011; Klein and Ronez, 2012), potentially exacerbating bone marrow platelet production deficits (François et al., 1997; Stéphan et al., 1999; Buyse et al., 2010). Furthermore, KPLA-related liver damage may cause TPO deficiency, which is crucial for platelet production and is primarily liver-derived, leading to decreased platelet counts (Lee-Sundlov et al., 2022). Infections can also trigger sialidase release, hydrolyzing sialic acid on platelets and thus inducing thrombocytopenia (Li et al., 2017).

Contemporary research suggests that the utility of transfusion or pharmacotherapy for infection-related thrombocytopenia may be circumscribed. The cornerstone of thrombocytopenia management is the resolution of the underlying infection, which is exemplified by prompt drainage in cases such as PLA, where puncture does not exacerbate bleeding risks even in the presence of coagulopathy (Wang et al., 2021). Adequate drainage and appropriate long-term antibiotic therapy, particularly with carbapenems such as meropenem, imipenem, and ertapenem, are beneficial for treating KPLA and can reduce associated mortality risk (Di Carlo et al., 2011; Chong et al., 2018; Di Carlo et al., 2019). However, for carbapenem-resistant Klebsiella pneumoniae, treatment options are limited, resulting in high mortality rates among ICU patients (Mammina et al., 2012; Boyd et al., 2017). Platelet count resurgence typically trails behind clinical improvement (Menard et al., 2019). A protracted absence of platelet recovery is often indicative of a more severe disease and correlates with adverse clinical prognoses. Additionally, the timelines for platelet count normalization observed in our study parallel those documented in sepsis and septic shock cases (Li et al., 2017; Menard et al., 2019).

Our study had some limitations. First, the inability to directly measure markers of platelet destruction and depletion led us to infer platelet consumption from the characteristic presentation of thrombophlebitis. Second, the heterogeneity in patient management across different hospital departments resulted in inconsistent treatment regimens, which could have influenced the outcomes. Third, detailed differential analysis of bone marrow findings was lacking, which was primarily due to the small number of KPLA patients at Shengjing hospital who underwent bone marrow aspiration. To overcome these limitations and corroborate the study findings, further research involving larger-scale, multi-center studies is recommended.

In conclusion, the identification of thrombocytopenia serves as a crucial marker for determining KPLA severity. Diminished platelet production within the bone marrow coupled with escalated consumption of platelets in the periphery likely constitute the primary pathophysiological underpinnings of thrombocytopenia in patients with KPLA.

## Data availability statement

The original contributions presented in the study are included in the article/supplementary material. Further inquiries can be directed to the corresponding author.

## Ethics statement

The studies involving humans were approved by the Ethics Committee of the Shengjing Hospital of China Medical University. The studies were conducted in accordance with the local legislation and institutional requirements. The ethics committee/institutional review board waived the requirement of written informed consent for participation from the participants or the participants’ legal guardians/next of kin because Patients’ identification remained anonymous and informed consent was waived due to the observational nature of the study.

## Author contributions

LC: Writing – original draft, Methodology, Investigation, Formal Analysis, Data curation, Conceptualization. HW: Investigation, Writing – original draft, Methodology, Formal Analysis, Data curation, Conceptualization. HW: Writing – original draft, Formal Analysis, Data curation. YG: Writing – original draft, Validation, Methodology. ZC: Writing – review & editing, Supervision, Resources, Methodology, Investigation, Funding acquisition, Formal Analysis, Data curation, Conceptualization.
